# Development and pre-testing of a family-centered digital intervention to help families know what to EXPECT from pediatric acute lymphoblastic leukemia therapy

**DOI:** 10.1007/s00520-026-10835-3

**Published:** 2026-06-15

**Authors:** Clara C. Hatch, Lydia Haupt Levy, Kara M. Kelly, Anna Revette, Marissa Krieger, Laura Moynihan, Natasha Carter, Rachel Selig, Lynda M. Vrooman, Justine M. Kahn, Katie A. Greenzang

**Affiliations:** 1 Dana-Farber/Boston Children’s Cancer and Blood Disorders Center, 450 Brookline Ave, Boston, MA 02215 USA; 2 Division of Population Sciences, Dana-Farber Cancer Institute, Boston, MA USA; 3 Department of Pediatric Oncology, Roswell Park Comprehensive Cancer Center, Buffalo, NY USA; 4 Division of Pediatric Hematology/Oncology, University at Buffalo Jacobs School of Medicine and Biomedical Sciences, Buffalo, NY USA; 5 Division of Pediatric Hematology/Oncology, Hasbro Children’s Hospital, Providence, RI USA; 6 Department of Pediatrics, Columbia University Irving Medical Center, New York, NY USA; 7 Harvard Medical School, Boston, MA USA

**Keywords:** Pediatric Oncology, Leukemia, Communication, Digital health, Patient education

## Abstract

**Purpose:**

Acute lymphoblastic leukemia (ALL), the most common pediatric malignancy, involves multi-year treatment with substantial outpatient care, which contributes to high parent/caregiver burden. Communication gaps and complex treatment regimens can leave families feeling unprepared for care of their children with ALL. To address this, we co-developed and pre-tested a digital health intervention supporting communication about what to expect during and after pediatric ALL treatment.

**Methods:**

Employing an iterative, stakeholder-driven process, we co-developed and pre-tested a family-centered interactive website, “EXPECT,” in two phases. Phase 1 included a secondary synthesis of stakeholder needed information and functionality (contextual inquiry), content creation, and website development, overseen by a multidisciplinary steering committee of clinical experts, patients, and families. In Phase 2 pre-testing, parents of patients with ALL rated the website’s acceptability and provided feedback during semi-structured interviews.

**Results:**

Synthesis of previous patient, parent, and provider interviews revealed key priorities for intervention content and design which were addressed in content creation and web development. Pre-testing demonstrated high acceptability (4.7/5). Participants highlighted EXPECT’s user-friendliness and innovative features such as the interactive treatment roadmap. Participants preferred to receive early access to the website, within weeks of initiating treatment, and anticipated using EXPECT throughout therapy and the post-treatment period, primarily on mobile devices.

**Conclusion:**

EXPECT co-development resulted in an acceptable digital health intervention to support families of children with ALL. EXPECT is being refined in response to feedback and will be tested in real-time treatment discussions in a multi-center pilot study to evaluate feasibility, use, and preliminary efficacy.

**Supplementary Information:**

The online version contains supplementary material available at 10.1007/s00520-026-10835-3.

## Introduction

A childhood cancer diagnosis requires families to absorb large amounts of complex information while under high stress [1]. Despite lengthy consent forms and extensive treatment discussions, communication gaps remain [2–4]. Many parents/caregivers feel inundated with data that is not presented in ways they need to prepare for the future or make decisions about their child’s care [5, 6]. Treatment regimens have grown more complex, and increasingly considerable care occurs outpatient and at home. High-quality communication and access to information throughout pediatric cancer treatment and survivorship can enhance parent knowledge and understanding, support treatment decision-making, foster trust in the healthcare team, and promote self-efficacy in managing care [7–9]. Digital health, or eHealth, tools are a promising strategy to provide tailored information and support to families facing pediatric cancer [10]. However, the rarity of pediatric cancer and the diversity of diagnoses and treatment approaches have limited the development of interventions specific to individual diagnoses and treatment plans.

Acute lymphoblastic leukemia (ALL), the most common pediatric malignancy, is treated with complex, multi-year therapy with excellent long-term outcomes [11]. The incidence of this diagnosis, and complicated treatment plan, provides an optimal model to develop and test interventions to support families in understanding what to expect from cancer therapy in the short- and long-term [7–9]. At diagnosis, pediatric oncology clinicians describe challenges conveying the copious necessary information about ALL treatment [12]. During therapy, poor adherence to the complex outpatient chemotherapy required of ALL therapy is a critical contributor to risk of relapse [13, 14]. Yet existing medication teaching is highly variable [15], and families struggle to follow administration guidelines and seek more educational resources [16]. After treatment completion, increased knowledge and preparedness for what comes next may play a critical role in engagement in long-term survivorship screening and care [17].

Qualitative interviews with patients with ALL and their families, and pediatric oncology clinicians, identified a need for an eHealth intervention that helps families synthesize treatment information and set realistic expectations for the care of children with ALL [1, 18]. Therefore, to improve family-centered communication and information about what to expect throughout childhood ALL treatment, we co-developed and pre-tested a digital health intervention called Expectations for Pediatric Cancer Treatment (EXPECT). EXPECT is an interactive website designed to provide comprehensive information to support families from diagnosis through survivorship in understanding ALL multi-phase treatment, anticipated symptoms and toxicities, home care responsibilities, medication administration, and potential long-term effects and survivorship care. In this way, it addresses core cancer communication functions of exchanging information, making informed decisions, and enabling patient self-management [19, 20]. EXPECT was designed to support patients and families receiving treatment as part of the Dana-Farber Cancer Institute (DFCI) ALL Consortium—a group of eight institutions conducting iterative cooperative group trials to improve treatment for ALL. Here we describe the user-centered, iterative design process and pre-testing.

## Methods

### Study design

**Fig. 1:**
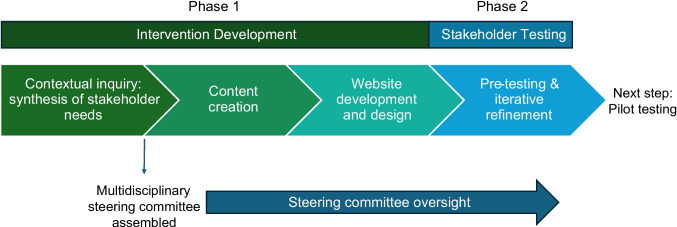
EXPECT development process

This study utilized a user-centered design [21], engaging patients, parents, and clinical experts in all phases of development and testing, and overseen by an interdisciplinary steering committee of pediatric oncology clinicians and patient families (Fig. 1). Phase 1 encompassed intervention development through three steps: (1) contextual inquiry including secondary synthesis of stakeholder needs from prior qualitative interviews [22], (2) content creation, and (3) website development and design. Phase 2 included pre-testing with key stakeholders to assess content, functionality, and acceptability prior to planned pilot-testing.

### Phase 1: Intervention development

#### Step 1: Contextual inquiry of previously conducted qualitative interviews

Previously conducted qualitative interviews with two distinct groups—patients and families, and clinical experts—were synthesized to identify core content areas and intervention approaches across varied stakeholders. Patient/family interviews included parents of patients actively receiving treatment (*n* = 12), parents of survivors (*n* = 10), and adolescent and young adult (AYA) survivors of pediatric ALL (*n* = 8) at the Dana-Farber Cancer Institute/Boston Children’s Hospital Cancer and Blood Disorders Center (DFCI/BCH) and treated on a DFCI ALL Consortium trial [1]. Healthcare providers representing multiple disciplines, including physicians (*n* = 5), nurse practitioners (*n* = 3), a nurse, a pharmacist, and a social worker who care for children with ALL at DFCI ALL Consortium sites including DFCI/BCH, Roswell Park Comprehensive Cancer Center, Hasbro Children’s Hospital, and Columbia University Medical Center were interviewed separately [18]. Detailed information on interview participants and approaches, interview guides, and comprehensive qualitative analysis was previously reported, and identified a need for a digital intervention [1, 18].

To determine the core required components of EXPECT across stakeholder groups and ensure it fit within current clinical practice, we conducted an amplified supplemental secondary analysis [23, 24]. We evaluated common themes across distinct participant groups (amplified), with special focus on intervention support needs (supplemental).

Team-based collaborative coding of initial study data was completed by the same team of qualitative researchers. This analysis focused on codes related to desired functionality and components of a supportive intervention. This subset of data was combined across groups into a single data set and re-reviewed. Building on the prior analysis, the data was re-coded deductively focusing on overlapping needs across groups. Themes were refined through iterative team-based discussion. Data analysis and management were supported by NVivo (QSR International, Version 1.4.1, 2021).

##### Steering committee (SC)

A multidisciplinary SC was assembled to oversee all subsequent phases. The SC included representatives with distinct areas of clinical expertise, from multiple DFCI ALL consortium sites, and patients and parents. Members were recruited from Step 1 interview participants, and all were employed or treated at DFCI ALL Consortium sites. The final SC included three parents of pediatric ALL survivors, one young adult ALL survivor, six clinicians from various sites and clinical roles across the DFCI ALL consortium (three physicians, two nurses, and one social worker), and members of the study team. The multidisciplinary SC convened regularly to review Step 1 interview themes, engage in content discussions, review website prototypes, verify alignment with stakeholder needs, and ensure medical accuracy.

#### Step 2: Content creation

The study team, with SC oversight, developed content to address key priorities outlined in Step 1 synthesis. Subcommittees of topic experts, including nurse navigators, a dermatologist, a pharmacist, survivorship experts, and a graphic designer developed content in areas of expertise.

Existing local and national resources were reviewed, and the study team and expert consultants adapted existing content where appropriate and developed new content as required using a mix of written, visual, and audiovisual displays. Best practices of risk presentation and communication accessible to those with variable levels of health literacy were utilized, including headers and bulleted lists to facilitate information retention. Concepts and scripts for educational videos were developed through a group writing process.

#### Step 3: Website development and design

The DFCI Digital Health Innovations Group assisted with website development, vendor selection and communication, and privacy and regulatory compliance throughout. The website was developed on a password-protected, HIPAA-compliant platform for use across devices, including personal computers, tablets, and smartphones, in collaboration with a vendor experienced in digital health intervention development.

### Phase 2: Stakeholder pre-testing

In keeping with best practices of digital health intervention design, pre-testing was conducted to determine acceptability and refine EXPECT prior to pilot-testing [25].

### Participants and procedures

Phase 2 pre-testing involved real-time use and navigation of EXPECT with parents of children actively receiving ALL treatment and parents of ALL survivors who were treated at DFCI/BCH and who received DFCI ALL Consortium treatment. Parents were recruited from December 2024 to June 2025 and purposively sampled to represent different phases of treatment, risk groups, and race/ethnicity. Some parents participated previously in Phase 1 interviews. Non-English-speaking parents and those whose child had relapsed or refractory disease were excluded.

Individual semi-structured interviews were conducted over Zoom or in-person by trained interviewers. Participants first reviewed a one-page handout introducing EXPECT with a broad visual overview of ALL treatment (Supplemental Material 1). Then, participants accessed and interacted with the website on multiple device types, including Mac and PC computers, tablets, and smartphones. Interviews followed a templated interview guide that prompted parents to “think out loud” as they engaged with the website based on three scenarios: receiving a new ALL diagnosis, undergoing treatment, and transitioning to survivorship care. They were encouraged to think of questions at each stage and to use the site to seek answers. Interviews lasted 45–60 min; participants received a $20 gift card in thanks for their participation.

### Quantitative measures

After the interview, participants completed a validated 6-item acceptability scale [26]. The acceptability scale assesses ease of use, enjoyability, helpfulness, satisfaction, time spent, and understandability rated on a scale of 1–5, where 1 denotes a negative evaluation, 3 neutral, and 5 a positive evaluation.

### Data collection and analysis

Detailed interviewer notes were taken during all interviews; interviews conducted over Zoom were audio recorded to supplement notes. Rapid analysis was performed through organizing and synthesizing notes within and across participants in a prefigured template to identify strengths and vulnerabilities of the usability, comprehensibility, and functionality of the intervention [27, 28]. We also aimed to detect technical issues and determine the ideal timing for introducing the website. User-testing interviews were conducted until meaning saturation was achieved and no new functional concerns were discovered [29, 30]. Acceptability scale single-item scores and summary scale scores were tabulated.

## Results

### Phase 1: Intervention development

#### Step 1: Contextual inquiry of previously conducted interviews

All participants sought a digital interactive resource to provide additional information and streamlined support throughout ALL treatment and into survivorship, specifically a website, paired with a printed information sheet introducing the website [1, 18]. AYA survivors largely deferred to their parents for treatment-related information, so a single parent-facing intervention was preferred.

**Fig. 2:**
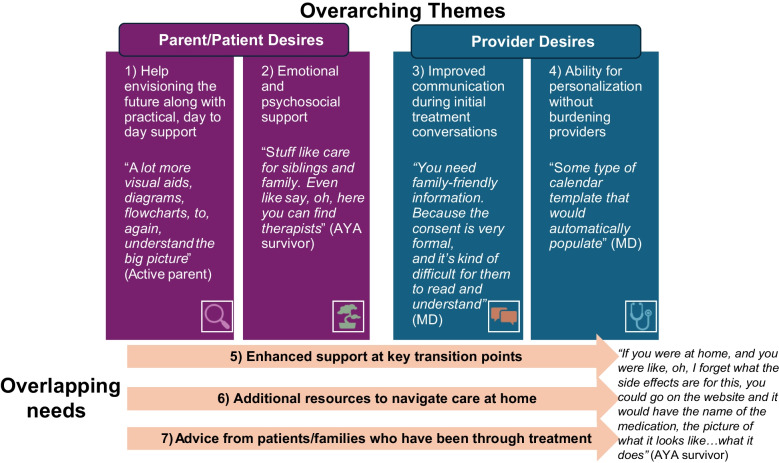
Patient, parent, and provider desired elements of intervention

Patients/families and providers had several overlapping priorities for website content and design, and some separate but aligned preferences by participant groups (Fig. 2). Parents and survivors valued: (1) “big picture” visual representations of treatment to understand what to expect and when, along with practical, logistical tips for day-to-day support, such as how to prepare for a day in clinic, and (2) psychosocial resources. Providers emphasized the need for (3) improved information support during initial treatment discussions with families, and (4) the ability to tailor information for different families or treatment risk groups. Both groups valued (5) enhanced support at key timepoints such as transitioning off treatment, and both sought (6) additional resources to support care at home. Providers also reinforced family preferences for (7) opportunities to connect with and learn from other patients and parents who have been through ALL treatment.

#### Step 2: Content creation

Themes identified in Step 1 (Fig. 2) generated the key content and website architecture to meet both family and clinician needs. Distinct content was developed or adapted and organized based on treatment stage (newly diagnosed, going through treatment, survivorship) and category (home care, emotional support, procedures). Information from existing vetted resources such as Blood Cancer United and the NCI Physician Data Query (PDQ) Cancer Information Summaries was adapted when available [31, 32]. New resources were created to address topics of major concern, such as steroid-related side effects.

#### Step 3: Website development and design

EXPECT was programmed on a password-protected, HIPAA-compliant digital platform. It is designed for use on a variety of devices (computers, tablets, mobile phones) to be accessible across settings, including in the hospital during inpatient stays, in the clinic for outpatient care, and at home.

##### Overview of EXPECT

**Fig. 3:**
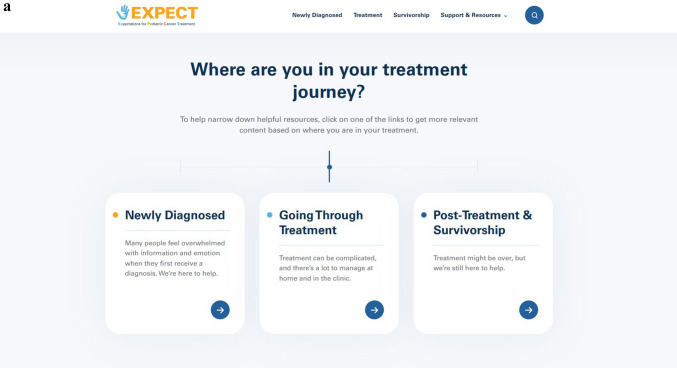

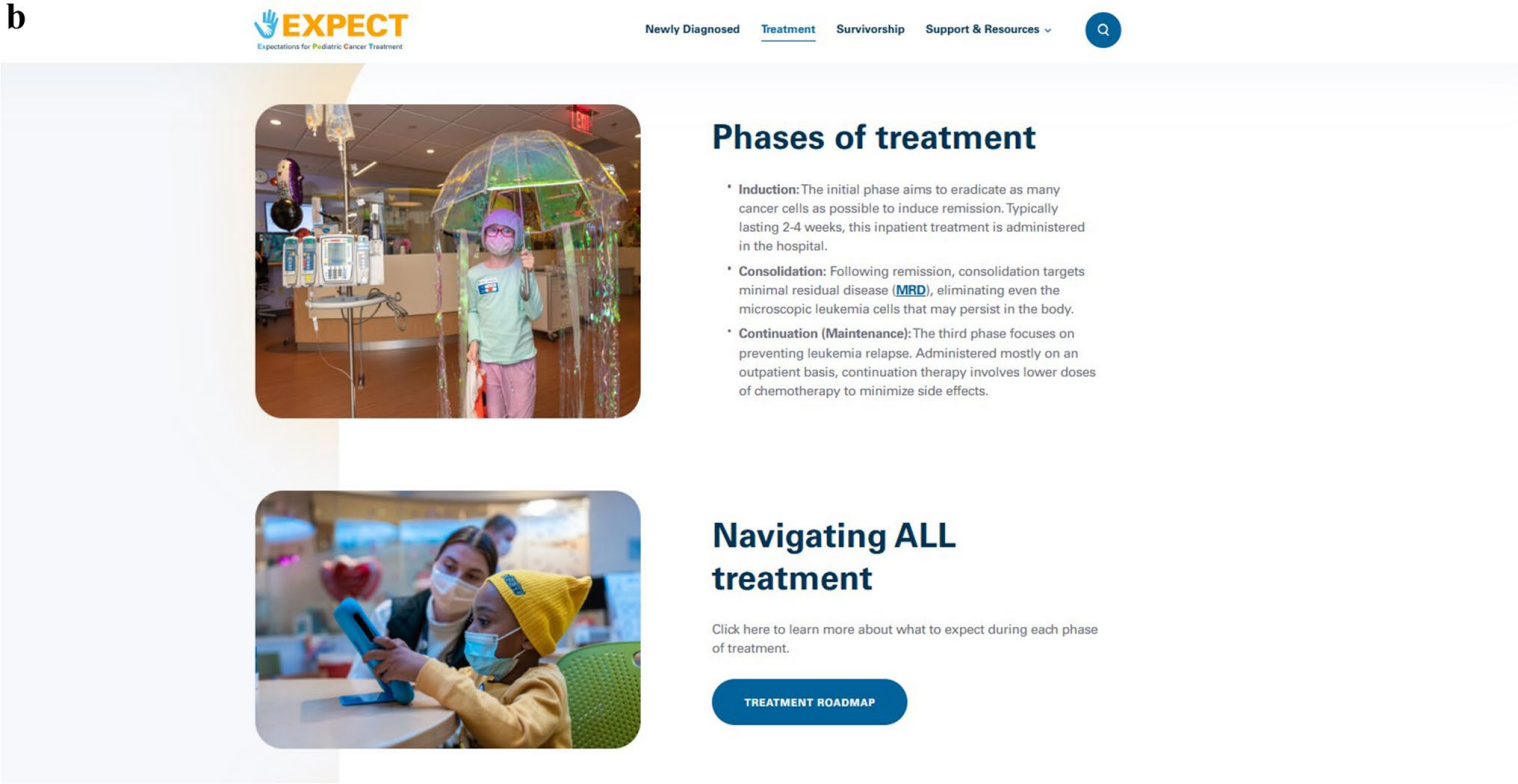
**a** EXPECT website homepage. **b** “Going through treatment” webpage

In recognition that patients and families in different phases of therapy require distinct information, the website begins with a choice-based format, allowing families to engage with information most relevant to their child’s current stage in treatment (newly diagnosed, going through active treatment, or post-treatment and survivorship), with the option to look ahead to the future when ready (Fig. 3). In this way, EXPECT contains guidance pertinent to each phase of therapy, but also allows families to review information that is immediately relevant, or to look far ahead, per individual preference. Directly responsive to the needs highlighted in Step 1 contextual inquiry, EXPECT includes: 1) an interactive treatment “roadmap” and treatment-phase-specific medication calendars, which hyperlink to teaching sheets detailing agent-by-agent mechanisms of action, side-effects, and potential toxicities [33], 2) brief informational and supportive videos of providers and families addressing core topics such as an overview of ALL treatment, and what to expect in clinic visits; 3) a searchable resource library addressing topics such as steroid management and sibling support, where additional resources can be added over time, and 4) a single-page handout previewing the website (Table 1). Upon entering the treatment roadmap webpage, users select their child’s treatment risk-group from a drop-down menu. As risk groups evolve over the course of treatment, users can change which treatment risk group they are viewing. Treatment roadmaps and medication calendars are pre-populated corresponding with DFCI ALL Consortium risk groups, creating a high level of specificity with minimal user input required. The digital materials clearly state that they are examples, and acknowledge that treatment plans may be modified or change over time.

**Table 1:**
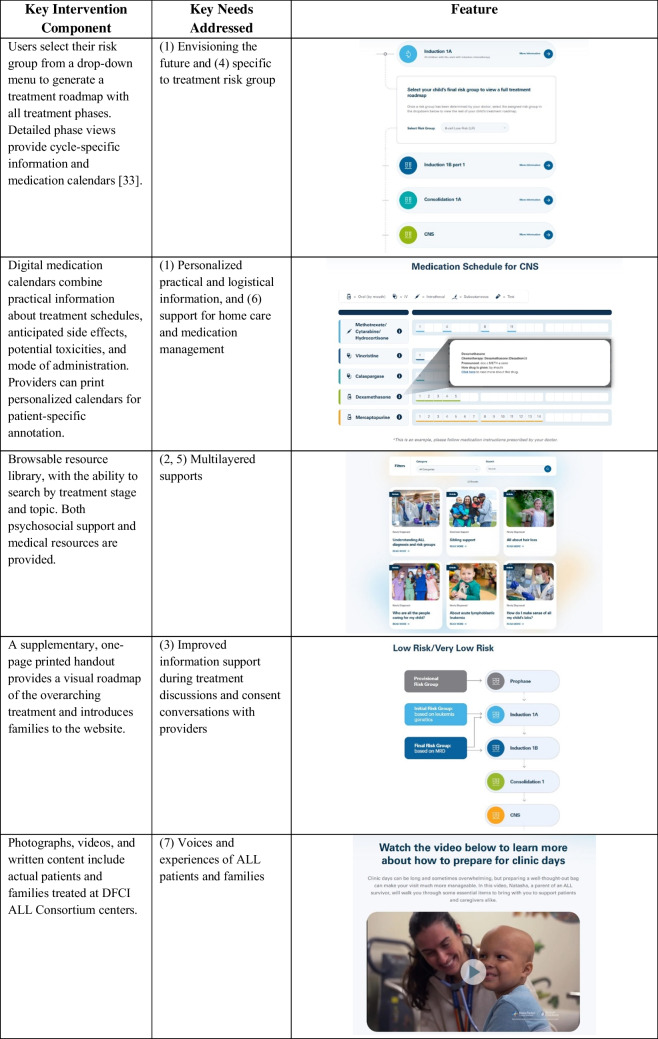
Core website features based on qualitative interview feedback

Comprehensibility, navigability, and applicability across the treatment trajectory were top priorities, as was the ability for users to seek more or less detailed information. Features such as collapsible lists and responsive design were implemented to support flexible engagement.

Another consideration was how and when to introduce EXPECT to families. In recognition of patient and family desires for “big-picture” visualizations and provider requests for additional informational support around the time of diagnosis, a handout depicting a visual roadmap of overarching ALL treatment paired with instructions on how to access the website was created to be shared with families after they consent to clinical trial treatment, to support but not supplant informed consent conversations (Supplemental Material 1).

### Phase 2: Stakeholder user testing

Pre-testing of EXPECT was conducted with ten parents of ALL patients, including six parents of patients actively receiving treatment and four parents of survivors. Most participants were female (80%) and white/non-Hispanic (80%). Patients of participating parents ranged from 2 to 15 years old; seven patients had B-ALL, two T-ALL, and one mixed phenotype ALL, ranging from low to high risk.

#### Quantitative acceptability

**Fig. 4:**
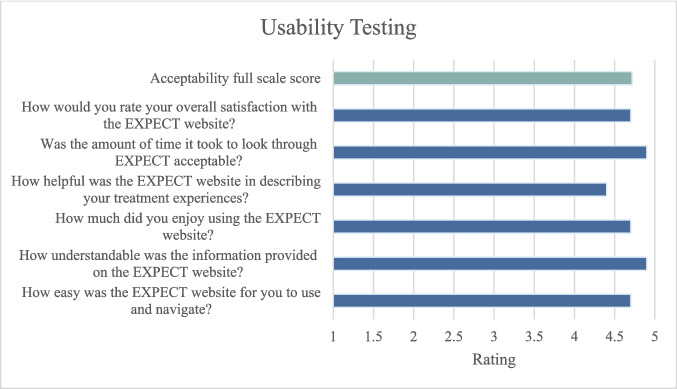
User acceptability testing results (N = 10). Each item is rated on a scale of 1–5, where 1 denotes a negative evaluation, 3 neutral, and 5 a positive evaluation

EXPECT was rated as highly acceptable, with an overall satisfaction rating of 4.7/5 (Fig. 4). The mean acceptability rating across all items was 4.7 (SD = 0.63, range 2–5). Comprehensibility and acceptability of time spent using EXPECT were the highest rated items (mean = 4.9/5).

#### Qualitative feedback

Pre-testing interviews similarly demonstrated appreciation for EXPECT, specifically website design, clarity, and personalization. Participants found information accessible without being overwhelming and appreciated the balance between emotional support and medical guidance. Participants valued the flexibility to engage with content in a variety of formats (videos, text, digital) and at different levels of detail. Parents whose child completed treatment were most interested in survivorship information and resources. However, all participants expressed interest in the “newly diagnosed” content, reflecting longitudinal interest in this information.

The interactive treatment roadmap and medication calendars were cited as standout features across participant groups. Parents valued the ability to explore information specific to their child’s treatment phase and risk group and filter out information not relevant to them. The roadmap was also seen as filling a critical gap by simultaneously providing information on day-to-day management and long-term considerations. The phase-specific medication calendars were a convenient and appreciated way to manage at-home administration and “look ahead” to upcoming treatment cycles.

Most participants indicated that they would primarily access EXPECT on a smartphone, particularly during inpatient stays or in waiting rooms, but appreciated optimizing EXPECT across platforms. Most recommended providing access to the website within the first month following diagnosis but emphasized that it should be available throughout treatment. One parent described the site as “giving you all the information you get in the first five days, but you can absorb it at your own rate.” Parents anticipated using EXPECT to review information after seminal treatment conversations and in support of home care, particularly when clinic visits become less frequent.

User-testing suggestions for modification primarily encompassed content expansion and minor clarifications. In response to feedback, guidance on steroid management was expanded, and additional resources on returning to school and financial support will be added prior to pilot-testing.

## Discussion

We developed a family-centered, novel eHealth intervention to support ongoing communication and information about what to expect during and after pediatric ALL treatment, called EXPECT. EXPECT was co-developed with clinical experts, patients, and families, and found to be highly acceptable in pre-testing with parents of children with ALL. The stakeholder-engaged development process ensured that various needs were addressed throughout, including those of parents and clinical experts across several DFCI ALL Consortium sites with distinct patient populations and resources. Participant feedback underscored the value of a resource introduced early in treatment that families can review at their own pace, with the ability to return to information over time and explore more in-depth subjects when ready.

We next plan to pilot EXPECT in real-time patient care to assess detailed feasibility, use, and acceptability during treatment and preliminary communication and knowledge outcomes. In the upcoming pilot, EXPECT will be introduced by the study team during the first weeks of ALL therapy. Given our targeted approach to key communication functions of information exchange, self-management, and care decisions [19, 20], we hypothesize that EXPECT may ultimately facilitate improved understanding of what to expect from therapy, decrease decisional regret, and support parents in complex home care.

EXPECT was developed to support families of patients enrolled on the upcoming DFCI ALL Consortium clinical trial, 25-001. The DFCI ALL Consortium is a well-established, multi-institution group that conducts iterative clinical trials for newly diagnosed patients with ALL at sites across the USA and Canada. By designing our intervention in alignment with a larger multi-institution clinical trial for the most common pediatric malignancy, we aimed to develop an intervention acceptable and relevant across the diverse patient populations and communication approaches of the eight DFCI ALL Consortium sites, yet also specific to a single disease and treatment approach.

While many digital health interventions exist for adults with cancer to support information provision, communication, and symptom management [34], and there are various eHealth interventions for parents of patients with chronic conditions [35], fewer digital health interventions are available for pediatric cancer [10]. This is partially due to the rarity of pediatric malignancies and the wide array of diagnoses and treatment approaches. Recently developed interventions provide broad information to support symptoms experienced across a variety of pediatric cancers [28, 36]. Yet, patients and families express a need for information tailored to their specific diagnosis, treatment plan, and anticipated toxicities [1, 37, 38]. EXPECT is directly responsive to the challenges and complexities of pediatric ALL care faced by parents [39], and the communication and information provision challenges faced by pediatric oncologists [12, 18, 40].

The specificity of EXPECT to support families facing a single treatment approach for a single diagnosis enabled us to develop highly personalized and relevant information across the disease course. The website includes several innovative features that can be tailored to individual information needs with minimal provider or family effort, notably the interactive treatment roadmap and phase-specific medication calendars to support home care and self-efficacy. Additional novel features include comprehensive information about both treatment and survivorship to reflect the full arc of treatment in a single source, the ability for participants to access information aligned with their preferred level of detail, and information in a variety of formats inclusive of videos, text, and infographics.

Tailoring EXPECT to a specific clinical trial contributes to limitations as well. Any updates to the trial protocol will need to be reflected in EXPECT, requiring regular maintenance to ensure comprehensive, up-to-date information. While EXPECT supports the DFCI ALL Consortium approach to treatment, several other regimens and trials exist for this disease. Additional limitations include that Phase 1 and 2 enrollment was limited to English-speaking participants, and pre-testing participants were drawn from a single urban academic center with limited racial/ethnic diversity. However, our collaborative approach of co-developing content and resources with patients and families, digital health specialists, and clinical experts from multiple sites serves to enhance the intervention’s credibility and relevance. In the upcoming multi-center pilot study, EXPECT will be tested during real-time care across multiple Consortium sites serving diverse patient populations with variable resource needs. Future versions will be linguistically and culturally adapted in additional languages to support more families.

Treatment for pediatric ALL is complex, placing a significant informational and emotional burden on families. EXPECT offers reliable, vetted information and guidance in one place to deliver tailored information and support parental self-efficacy. It is novel in its specificity and minimization of workload for families and clinicians and was found to be highly acceptable in pre-testing. Next steps include incorporating user feedback to expand the resource library, and adapting to reflect the updated treatment roadmaps in DFCI 25-001. Then, EXPECT will be pilot-tested in a multi-center study to evaluate real-time use. If feasible and effective, this intervention could become standard-of-care for families newly diagnosed with ALL treated with the DFCI ALL Consortium approach, and could be adapted to support information delivery and home care across different pediatric malignancies to help parents feel prepared and empowered.

## Supplementary Information

Below is the link to the electronic supplementary material.

ESM 1 (PDF 281 KB)

## Data Availability

No datasets were generated or analysed during the current study.
