# Supplementary material for: Development and pre-testing of a family-centered digital intervention to help families know what to EXPECT from pediatric acute lymphoblastic leukemia therapy
Source: Support Care Cancer. 2026 Jun 15;34(7):658. doi: 10.1007/s00520-026-10835-3 (PMC13269404; doi:10.1007/s00520-026-10835-3)
Supplement: Supplementary file 1 — (PDF 281 KB) [file 520_2026_10835_MOESM1_ESM.pdf]

EXPECT

Expectations for Pediatric Cancer Treatment

Welcome to **EXPECT**! A resource to support parents, caregivers, and patients facing childhood Acute Lymphoblastic Leukemia (ALL).

Scan the QR code or go to expect.dana-farber.org to learn more and enter the password [redacted].

Low Risk/Very Low Risk

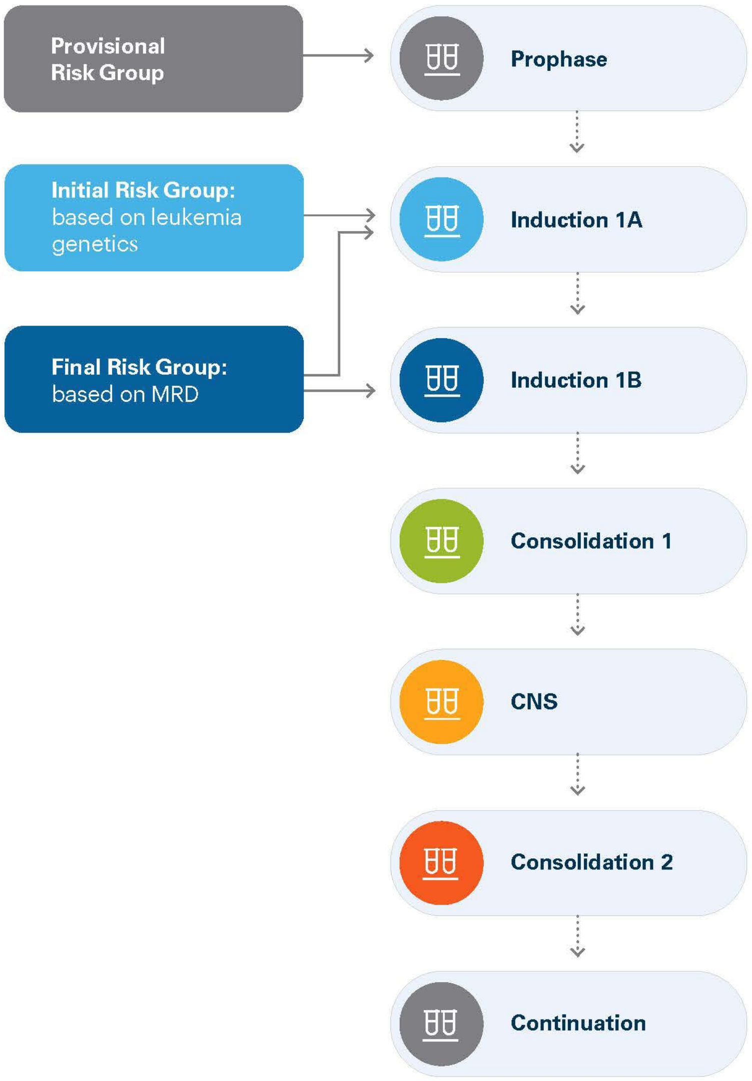

Copyright © 2024 Dana-Farber Cancer Institute, Inc. and The Children's Hospital Corporation d/b/a Boston Children's Hospital All Rights Reserved.

Dana-Farber Cancer Institute | Boston Children's

Dana-Farber/Boston Children's Cancer and Blood Disorders Center
